# Supporting Decision-Making About Patient Mobility in the Intensive Care Unit Nurse Work Environment: Work Domain Analysis

**DOI:** 10.2196/41051

**Published:** 2022-09-27

**Authors:** Anna Krupp, Linsey Steege, John Lee, Karen Dunn Lopez, Barbara King

**Affiliations:** 1 College of Nursing University of Iowa Iowa City, IA United States; 2 School of Nursing University of Wisconsin-Madison Madison, WI United States; 3 Department of Industrial and Systems Engineering College of Engineering University of Wisconsin-Madison Madison, WI United States

**Keywords:** clinical decision-making, early ambulation, intensive care unit, nursing, qualitative research, cognitive work analysis

## Abstract

**Background:**

Patient mobility is an evidenced-based physical activity intervention initiated during intensive care unit (ICU) admission and continued throughout hospitalization to maintain functional status, yet mobility is a complex intervention and not consistently implemented. Cognitive work analysis (CWA) is a useful human factors framework for understanding complex systems and can inform future technology design to optimize outcomes.

**Objective:**

The aim of this study is to understand the complexity and constraints of the ICU work environment as it relates to nurses carrying out patient mobility interventions, using CWA.

**Methods:**

We conducted a work domain analysis and completed an abstraction hierarchy using the CWA framework. Data from documents, observation (32 hours), and interviews with nurses (N=20) from 2 hospitals were used to construct the abstraction hierarchy.

**Results:**

Nurses seek information from a variety of sources and integrate patient and unit information to inform decision-making. The completed abstraction hierarchy depicts multiple high-level priorities that nurses balance, specifically, providing quality, safe care to patients while helping to manage unit-level throughput needs. Connections between levels on the abstraction hierarchy describe how and why nurses seek patient and hospital unit information to inform mobility decision-making. The analysis identifies several opportunities for technology design to support nurse decision-making about patient mobility.

**Conclusions:**

Future interventions need to consider the complexity of the ICU environment and types of information nurses need to make decisions about patient mobility. Considerations for future system redesign include developing and testing clinical decision support tools that integrate critical patient and unit-level information to support nurses in making patient mobility decisions.

## Introduction

### Background

Patient mobility is a critical intervention for intensive care unit (ICU) patients because hospital-acquired functional decline or a new loss in independently completing activities of daily living is a common complication of hospitalization, occurring in at least 50% of patients who require intensive care [1,2]. Mobility is a daily, progressive physical activity intervention for physiologically stable hospitalized patients, beginning with exercises in bed, transferring to a chair, and advancing to walking. International ICU guidelines recommend beginning mobility interventions in the ICU and continuing throughout hospitalization to maintain patients’ physical functioning during hospitalization as a standard of care [3,4]. Prolonged bedrest is a modifiable risk factor associated with functional decline, and increasing mobility in patients in the ICU is a priority, as patients who develop functional decline are at increased risk for prolonged hospitalization, discharge to a skilled nursing facility, readmission, inability to return to work, and premature death [5-9]. Despite increasing research on the benefits of ICU-based patient mobility interventions, routine implementation of mobility into clinical practice is limited.

Possible explanations for the lack of widespread implementation of mobility interventions into routine practice may include the fact that mobility guidelines are complex and challenging to implement and require thorough assessment to determine a patient’s stability and mobility status, team training and coordination to maintain safety and monitor equipment, and sufficient physical space for the patient, team, and equipment to complete activities. Mobility fits within several clinical practice domains, with physical therapists (PTs) or registered nurses (RNs) being most frequently involved in planning and implementing ICU mobility interventions [10]. RNs and PTs face multiple barriers when trying to implement mobility interventions. The major barriers to ICU-based mobility interventions have been categorized by previous researchers into the following 4 domains: patient (eg, physiologic instability, sedation, patient safety concerns), clinician (eg, inadequate training, workload, safety risk), process (eg, lack of coordination, unclear protocols), and organizational (eg, lack of mobility culture, competing priorities) [11-13]. Strategies to address barriers have included use of structured quality improvement models to identify and target local barriers [14], RN-initiated mobility protocols to standardize patient assessment and goal setting [15], and focused interdisciplinary communication and collaboration [16,17]. However, these resources and approaches have not spread widely. A limitation to mobility protocols is the poor usability given the temporal and cognitive demands associated with RN workflows.

Clinical decision support (CDS), or tools that integrate guideline recommendations with patient-specific information at the right time and at the point of care, has the potential to support the implementation of mobility guidelines into practice. CDS interventions are particularly relevant in ICU settings due to the large amounts of electronic health record (EHR) data generated and the need for time-critical decisions. CDS interventions to identify risk for clinical deterioration [18] and sepsis recognition [19] have led to improved intervention timeliness and decreased patient mortality. CDS data visualization has been shown to improve timeliness for delivering evidence-based sedation and mechanical ventilation practices [20], yet CDS to support mobility practice does not exist. In addition, CDS has focused primarily on medical decision-making, and less research has targeted decision support for acute care RNs [21]. CDS can provide data visualizations, alerts, reminders, and decision support to augment clinicians in complex processes and support coordinated care delivery [22]. However, without understanding the environment within which decisions are made, CDS may not be successfully developed and implemented [23].

We propose examining mobility decision-making using human factors methods to understand the work environment in which mobility decisions are made. Human factors methods aim to understand interactions between humans and elements of a system to optimize outcomes [24,25]. The ICU setting is a complex and dynamic environment with high levels of technology, uncertainty, time pressure, and interprofessional teamwork. Therefore, a naturalistic decision-making model is relevant for exploring the process of making decisions while appreciating the complexity of the ICU setting [25]. Because nurses provide the greatest amount of direct patient care, are responsible for patient monitoring and care coordination, and determine the patient’s mobility intervention (eg, standing, walking), we aim to understand nurse decision-making regarding patient mobility. The complexity of ICU nursing work includes multiple types of demands, such as physical, emotional, and cognitive, with nurses often making decisions under time pressure and unpredictability [26]. Understanding the environment in which ICU nurses make decisions, coordinate, and implement interventions is needed to guide the development of future interventions aimed at implementing and sustaining ICU mobility programs.

### Cognitive Work Analysis

One approach that has been used effectively to study real-world decision-making and inform informatics-based interventions in health care is cognitive work analysis (CWA). CWA is a methodological 5-phase framework that includes analytic tools used for systematically identifying different constraints or limitations in a work environment [27]. A fundamental concept of CWA is understanding constraints that result from the work environment, as they influence behavior and actions [28,29]. CWA uses a formative approach to analyzing the work environment. In contrast to analyzing how work should be done or how work is actually done, CWA describes principles of the work environment that are necessary for success and the range of ways that work is accomplished [27]. From this perspective, the work analysis identifies possible actions and requirements for those in a work environment to behave in new ways.

In addition, results of CWA inform system redesign and support developing decision support tools that offer flexibility in complex and unpredictable settings [29]. CWA has been applied in multiple health care settings, including ICUs, to understand complex settings and has informed design of new displays and decision support tools [30]. In summary, the theoretical approach of CWA aligns well with the current clinical challenge of implementing mobility interventions, as patients in the ICU are heterogeneous, there are multiple ways to achieve mobility interventions within a complex ICU work environment, and this analysis facilitates our long-term goal to develop CDS.

### Work Domain Analysis

Work domain analysis (WDA) is the foundational phase of CWA and focuses on understanding work environment purposes, constraints, and how the purpose(s) are achieved within the constraints of the work environment [27,31]. A result of the WDA is an abstraction hierarchy model, which provides a visual representation of the structure and functions of the work environment at different conceptual levels with means-ends linkages between levels. Table 1 summarizes the 5 abstraction levels. Each level provides a different perspective of the work environment; a linkage to a higher level describes *why* something exists and a linkage to a lower level describes *how* a purpose or function exists in the work environment.

**Table 1:** Abstraction hierarchy levels used for work domain analysis.

Abstraction level	Description	Example
Functional purpose	Reason why the work environment exists	Why does the ICU^a^ exist?
Values and priorities	Criteria to assess how well the work environment is performing its purpose	How do we know the ICU is achieving its purposes?
Purpose-related functions	High-level functions needed to support the values and priorities	What functions must be performed in the ICU to achieve its values and priorities?
Object-related processes	Describes what processes the objects in the work environment support	What are the functions of the resources in the ICU?
Physical objects	Objects within the work environment	What physical resources are in the ICU?

^a^ICU: intensive care unit.

Despite increasing research on the benefits of ICU-based patient mobility interventions, routine implementation of mobility guidelines into clinical practice is inadequate. A greater understanding of the environment in which ICU nurses make patient mobility decisions is needed to inform the development of future interventions. Therefore, the aim of this study was to apply WDA to develop an understanding of the complex ICU work environment and identify constraints as they relate to nurses carrying out patient mobility interventions.

## Methods

### Study Design

In this descriptive study, WDA was used as an approach for data collection and analysis. A WDA was conducted using data from multiple sources and followed an iterative process of data collection and model development. The scope of the WDA was the ICU work environment in the context of nurses carrying out patient mobility interventions.

### Setting

The study was conducted in 2 adult ICUs at 2 different health systems in a Midwestern US city. The study ICUs were chosen because they had established interdisciplinary mobility programs. Both ICUs had implemented protocols for managing pain, agitation, delirium, and immobility, which included orders for nurses to advance patient mobility as tolerated and PT orders dependent upon the medical team. Mechanically ventilated patients were routinely mobilized out of bed to the chair by nurses and often walked with PTs or RNs before ICU discharge. Both sites had a well-established safe patient handling and mobility program with patient lift equipment provided in the unit and staff training in safe patient handling. In addition, both units had similar nursing characteristics (eg, nurse staffing, unit leadership, shift length) and each organization had obtained Magnet Recognition for high-quality nursing care. Site 1 was a 24-bed closed-model ICU in an academic tertiary care center, and site 2 was a 12-bed open-model ICU in an academically affiliated Veterans Administration hospital. Although site 2 had slightly lower patient acuity, both units routinely admitted a range of medical and postsurgical patients.

### Data Sources

Multiple data sources were chosen to understand the ICU work environment within the perspective of mobility practice.

Organizational policies and published mobility guidelines were reviewed for relevance. Organizational policies were provided by nurse leaders at each site, and mobility guidelines were retrieved from PubMed. The following search terms were used: “guideline,” “intensive care units,” and “early mobility.” One researcher (AK) reviewed each document and extracted information related to the abstraction hierarchy levels.

Nurse observations (N=4, 32 hours total) were conducted at site 1 by 2 researchers (AK and another researcher) during normal routines of the first 4 hours of 2 separate shifts using a paper data collection tool. Both researchers observed the same nurse to capture communication, workflow, assessment, and coordination information to inform interview questions. Each nurse was interviewed within 1 week following the final observation.

Semistructured interviews (N=20) were conducted using an interview guide (Multimedia Appendix 1). Participants were asked to define mobility, describe case examples of mobilizing routine and complex patients, identify information used to make mobility decisions, barriers to mobility, and strategies used to overcome barriers. In addition, interviews with observation participants also included tailored questions based upon observation findings. For example, after one participant was observed using the EHR, an interview question was added to understand the specific information the participant was seeking. All interviews were conducted by 1 primary researcher (AK), audio recorded, and transcribed verbatim. Interviews lasted 45 to 60 minutes and were conducted in a private office at the nurse’s place of work.

### Participants

Nurses with 6 months or greater of current ICU experience and working 20 hours or more each week were eligible to participate. A purposive sampling method was used to select nurses with a range of experience while including expert nurses who routinely engaged patients in mobility activities. Participants were recruited via advertisement and recruitment materials in the unit and by nurse managers providing contact information for expert nurses with experience mobilizing patients.

### Ethics Approval

The University of Wisconsin-Madison institutional review board and the Madison Veterans Administration Research and Development Committee approved the study (approval #2016-1389).

### Analysis

Data analysis and abstraction hierarchy development were conducted iteratively with the data collection process. Nurse observation data were summarized, and follow-up interview questions were generated after each observation. Interview data were coded using an inductive approach. Two investigators, one with expertise in critical care nursing (AK) and the other in hospital ambulation interventions and qualitative methods (BK), analyzed the interview data using inductive content analysis [32]. Investigators individually performed open coding line by line by breaking down the data and assigning labels to identify preliminary key thoughts or concepts. Together, the research team grouped labels that were related to each other by content or context into subcategories. Subcategories were then collated into higher order main categories. Dedoose software (SocioCultural Research Consultants) was used for data management [33].

The abstraction hierarchy was developed by systematically and iteratively synthesizing data extracted from document review and subcategory and category codes from interview data. Steps from Naikar’s [31] 9-step method were used to develop the abstraction hierarchy. The first 5 steps describe decisions to consider before beginning the analysis and include defining the purpose and boundaries of analysis, identifying project and work domain constraints, and identifying sources of information for the analysis. For the purposes of our analysis, the boundaries of analysis were the ICU system, and interview information was limited to nurse participants. Our purpose focused on patient mobility work in the ICU system. Steps 6 through 8 consisted of iterative development of the abstraction hierarchy using available sources of information (eg, documents), special data collection (eg, observations, interviews), and domain expert review. The final step is validating the abstraction hierarchy. The initial abstraction hierarchy was developed based on data extracted from organizational mobility policies and published mobility guidelines. For example, documents described functions of team members, processes, and equipment available for mobility. The abstraction hierarchy was refined with data from interviews to include additional description and establish means-ends linkages between levels. The research team routinely discussed coding and refined the abstraction hierarchy. Later-occurring interviews at site 1 and all interviews at site 2 also included a review of the abstraction hierarchy. Participants were asked to identify if there were any missing, unclear, or redundant categories. The model was updated based on participant responses.

## Results

### Overview

Twenty nurses participated in the study (site 1=15, site 2=5). Participants had a range of 4 to 15 years of ICU experience, with a median of 7.8 years (Table 2).

**Table 2:** Demographic characteristics of participants (N=20).

Characteristic		Value
RN^a^ experience (years) median (IQR)		10.5 (6.6-16.4)
ICU^b^ experience, (years) median (IQR)		7.8 (4-14.8)
**Highest degree, n (%)**		
	Associate degree	1 (5)
	Bachelor’s degree	17 (85)
	Master’s degree	2 (10)
	Critical care certification	7 (35)

^a^RN: registered nurse.

^b^ICU: intensive care unit.

### Work Domain Analysis

**Figure 1:**
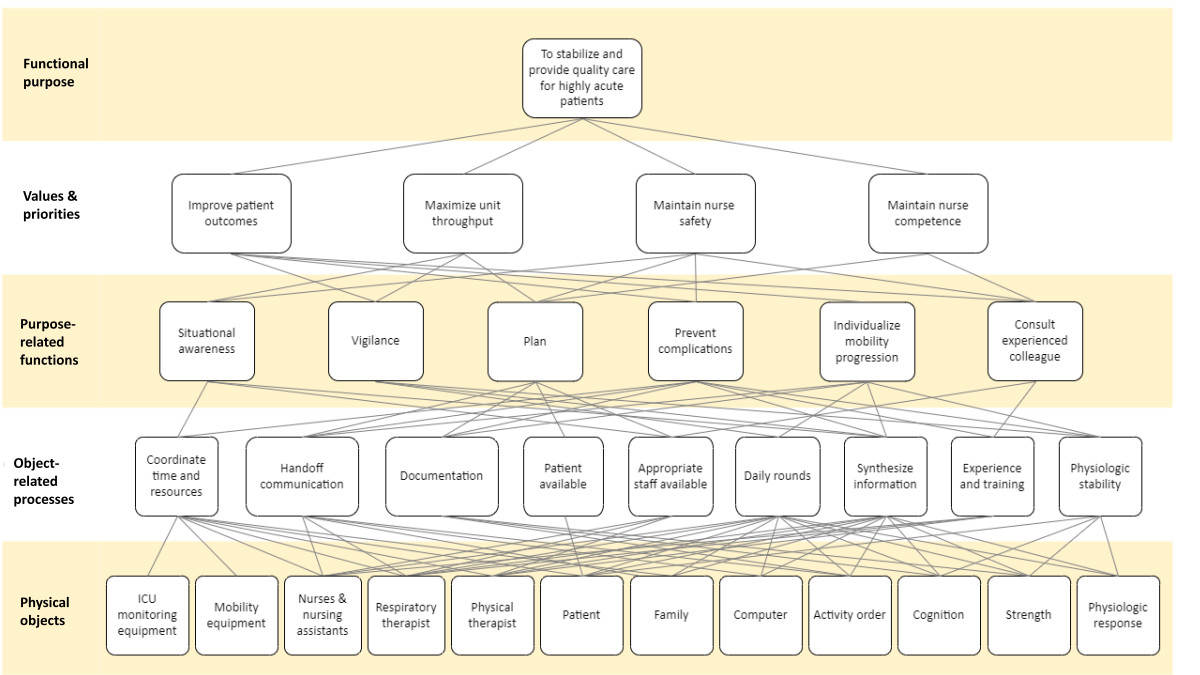
Abstraction hierarchy describing ICU work environment within the context of nursing implementing patient mobility. ICU: intensive care unit.

Results of the WDA are presented by abstraction hierarchy level and shown in part in Figure 1. Table 3 summarizes example category codes and representative quotes from the qualitative analysis that were used in developing the abstraction hierarchy. Results are presented by abstraction level.

**Table 3:** Sample codes and representative quotes by abstraction level.

Sample code by abstraction level			Representative quotes
**Functional purpose**			
	Stabilize	“There are some patients that are so critical that you really can’t move them, but I would say that is a small portion.” [RN^a^ 6]	
	Quality	“… mobility is a huge factor in getting somebody home because even if their illness has passed, if they’re not strong enough to take care of themselves they can’t go home.” [RN 2]	
**Values and priorities**			
	Patient outcomes	“We’ve gotten so safety oriented for fear of people falling that it’s hard sometimes to find a balance because even if a person seems totally alert and doesn’t have a lot of tubes, I think we’re still so scared that they’re going to fall.” [RN 11]	
	Nurse safety	“I’m not going to put myself in a situation or someone else in a situation where we’re going to get hurt… I have to protect myself.” [RN 13]	
	Throughput	“We’re moving people in and out, getting people to procedures, people are coming and going from everywhere.” [RN 4]	
**Purpose-related functions**			
	Vigilance	“If I come on a shift and they were moving up and down on the pressors all night or unstable in their heart rate, then I probably wouldn’t get them out of bed until at least the afternoon, so 4 to 6 hours of stability.” [RN 10]	
	Situational awareness	“We were getting 2 sick admissions, so I had to get him back to bed a little earlier than I wanted to.” [RN 3]	
	Individualize	“I want to know how they get up, with what equipment, and how many people do they need?” [RN 6]	
**Object-related processes**			
	Coordination	“About 20 minutes before that time I just started getting things together.” [RN 7]	
	Availability	“If it [a procedure] is not scheduled it can go one of two ways, you either leave them in bed until it happens, or you just get them up and hope and pray they don’t come right away.” [RN 1]	
	Appropriate staff available	“There are some patients, especially if we are talking about walking for the first time, that I will partner with physical therapy and not necessarily feel comfortable being the first person to stand them.” [RN 12]	
**Physical objects**			
	Human resources	“I usually need 2 to 3 people depending upon how many lines they have, if they’re intubated, if we need to pull the bed out of the room…” [RN 1]	
	Mobility equipment	“Walkers are a big problem because we have to order them up, they’re big, and somethings they take time to come [to the bedside].” [RN 6]	
	Computer	“I’d like to look more in the notes and see some progress, but I feel we don’t have time to do that.” [RN 14]	

^a^RN: registered nurse.

#### Functional Purpose

The functional or overall purpose of the ICU is to initially stabilize patients experiencing life-threating illness or injury and to provide high-quality care that improves outcomes for critically ill patients.

#### Values and Priorities

Four values and priorities were identified: improve patient outcomes, maintain nurse safety, maintain nurse competence, and maximize unit throughput. Most nurses described organizational priorities that focused on improving patient quality and outcomes, such as preventing patient falls. Moreover, staff safety and maintaining competency were promoted, as most participants described organizational training and resources available to nurses for safely assisting patients with movement. Maintaining ICU throughput was an additional priority, as most nurses described the routine, rapid turnaround of admitting highly unstable patients, stabilizing patients, and then transferring patients as soon as they met criteria for a lower level of care.

#### Purpose-Related Functions

Six purpose-related functions were identified, which describe how the values and priorities are achieved.

1. *Situation awareness* is recognition of unit activities and resources. Nurses described the need to assist with other unstable patients as a priority over patient mobility and increased activity on the unit limiting staffing resources to assist with mobility.
2. *Vigilance* is monitoring a patient’s stability over the work shift. For example, participants described watching patient vital signs on the bedside monitor while assisting them with repositioning in the bed or assessing for improvement in ability to follow directions over time. Multiple shifts with a patient provided nurses with more time to know the patient. Nurses stated that they felt more confident to progress mobility on the second day of providing care, based upon their assessment of how the patient tolerated a lower level of mobility the day prior.
3. *Plan* is the process of preparing for mobility interventions. Participants described multiple barriers that required clarification or adaptation before progressing mobility interventions. For example, nurses described time spent clarifying mobility orders with the medical team or asking family members about the type of assistive equipment a patient used prior to admission.
4. *Prevent complications* is implementing interventions to avoid harm. For the most severely ill patients, when short-term survival is not known, nurses identified that the priority is to achieve and maintain physiologic stability. As patients stabilize, participants described implementing progressive mobility interventions to prevent functional decline.
5. *Individualize mobility progression* is the process of assessing and determining the mobility goal for the shift. For patients that had not yet gotten out of bed in the ICU, nurses described spending considerable time assessing and synthesizing patient information to determine if the patient would tolerate out-of-bed mobility.
6. *Consult experienced colleague* is identifying and communicating with another health care professional to inform the daily mobility goal. In some situations, nurses described talking with a nursing assistant to learn how the patient tolerated walking during a prior mobility session, whereas in other situations, nurses described talking with a PT for medically complex patients with weakness or unsteady balance, or it being their first time out of bed after bed rest.

#### Object-Related Processes

Nine object-related processes were identified and are presented in Table 4. The object-related processes include the following: coordinate time and resources, handoff communication, daily rounds, documentation review, patient availability, staff availability, information synthesis, experience and training, and physiologic stability. These object-related processes describe how purpose-related functions are achieved and why physical objects exist in the work environment.

**Table 4:** Object-related processes identified in the abstraction hierarchy.

Object-related process	Description
Coordinate time and resources	Planning and organizing when mobility interventions occur in relation to patient needs, status of unit, and availability of human and equipment resources
Handoff communication	Information exchanged during nurse shift report
Daily rounds	Opportunity for various health care providers to discuss patient assessment, plan, and goals of care
Review documentation	Data and communication in the EHR^a^ that convey information about patient mobility, such as level and tolerance of previous mobility event
Patient available	Awareness of patient’s daily schedule included planned interventions, such as dialysis, and unanticipated events, such as a bed side procedure
Appropriate staff available	Matching patient needs as it relates to weakness, instability, and/or equipment with health care team member(s). For example, ensuring the respiratory therapist is available to assist with walking a patient requiring mechanical ventilation
Synthesize information	The process of analyzing information from multiple sources to individualize mobility progression
Experience and training	Training or experience with psychomotor skills, such as body positioning, body mechanics, and use of patient handling equipment
Physiologic stability	The ongoing assessment for changes in a patient’s physical status in relation to organ support required and evaluation of tolerance to changes in position or movement

^a^EHR: electronic health record.

#### Physical Objects

Twelve physical objects were identified and were grouped into components of human resources, equipment, and information sources. Physical objects describe how the object-related processes are implemented. Participants described human resources, primarily nursing assistants, other nurses and PTs, and mobility equipment, such as a walker, as necessary to support mobility interventions. Nurses used information from multiple sources to establish a mobility goal for the shift. Nurses described comparing current patient assessment information (eg, strength, cognition, and physiologic response to movement) and data from bedside monitors (eg, hemodynamic, respiratory, or neurologic values) to previous values to assess stability. Nurses also relied on peers during shift handoff reports and on patients or family members for verbal information.

#### Means-Ends Linkages

Means-ends linkages were identified between levels in the abstraction hierarchy and are illustrated in Figure 1 as connecting lines between abstraction hierarchy levels. The connections provide understanding between items, as moving from the top to a lower level in the abstraction hierarchy describes *how* the concept is achieved or carried out and moving from the bottom to a higher level in the abstraction hierarchy describes *why* a concept exists. For example, the object-related process of synthesizing information is connected to 10 physical objects, illustrating how nurses seek information from a variety of sources to inform mobility decision-making. Synthesizing information is also linked to 4 higher-level purposes, indicating why nurses integrate both patient and hospital unit information when making mobility decisions.

## Discussion

### Principal Findings

Our WDA describes the complexity of the ICU work environment within the context of nurses carrying out patient mobility interventions. The abstraction hierarchy depicts multiple high-level priorities that nurses balance, specifically, providing quality, safe care to patients while helping to manage unit-level throughput needs. Connections between levels on the abstraction hierarchy describe how and why nurses seek patient and hospital unit information to inform mobility decision-making. The WDA identifies several opportunities for technology design and future study at the nurse level and unit level to support nurse decision-making about patient mobility.

One key information need is to provide assessment information about patient stability to nurses. Nurses in this study described assessing the patient over the course of a shift or several shifts to individualize mobility progression, and nurses described information sources they used to determine patient stability for mobility. To improve the efficiency for nurses in seeking information about mobility decision-making, a trended display of patient stability for physiologic metrics that nurses currently use (eg, vital signs, respiratory indicators, sedation level) from the EHR may support decision-making. A second key information need is to provide historic patient mobility information to nurses. Participants described using information about the patient’s past mobility activity to inform their planning for the shift and looking for this information in multiple places. There are valid numerical scales to quantify mobility status and to efficiently communicate the highest level of activity a patient has achieved [34,35]. A current mobility value and trended display of the patient’s previous mobility progress or decline added to the EHR may support decision-making, as nurses in this study routinely sought out information about the patient’s previous mobility and response.

We have identified information that may be amenable to technology-based interventions to support nurse decision-making for mobility in the ICU. Studies using WDA-informed information displays have demonstrated improved ability for ICU and emergency room nurses to detect patient changes when compared to use of the existing EHRs [36,37]. From our WDA, we identified a need for indicators of patient stability (or instability) using trended patient data and indicators for mobility status. Future research on the feasibility and acceptability of prototypes that communicate this clinically meaningful information is needed.

Findings from the WDA also demonstrate opportunities and recommendations for unit-level information needs. Using technology interface design to organize relevant patient and unit information may offer several benefits. For example, displaying patient and unit information simultaneously may assist with limited resource allocation by prioritizing patients who display a greater need for mobility, such as a patient that has a greater duration of immobility. A unit display may also support a unit culture of accountability for mobility progression, as each patient’s mobility status is visible to the ICU team. A unit-based display or dashboard of real-time EHR data for promoting guideline-based mechanical ventilation care has been found to improve interprofessional care coordination, communication, and patient outcomes [20]. Future work is needed to develop and test visualizations for communicating guideline-based mobility care within the ICU.

### Limitations

This study should be considered within the context of several limitations. First, the study was conducted in 2 medical-surgical ICUs at 2 academic medical centers. Therefore, our findings may not be generalizable to all ICUs, but we expect the findings to be transferrable to similar settings. Differences in patient population, unit culture or available resources, ICU type, such as specialty surgical ICUs or community-based ICUs, may change work environment barriers. In addition, both study ICUs had experience with implementing mobility interventions. Work environment constraints and nurse decision-making might look different depending upon the status of implementing mobility practices, unit culture, teamwork, and ICU resources. Finally, the study focused on the work environment from the perspective of nurses. Although nurses described communication and coordination with other health care providers, future work is needed to include data from patients, family members, and additional team members in the work environment. The WDA is by no means a complete representation of the ICU work environment within the context of mobility interventions. Future work should expand the analysis to other layers of CWA.

One goal of the current study was to inform future system design to support the nurse in progressing patient mobility interventions. Therefore, our focus was on possible improvements in the work environment, such as information visibility, that could influence prototype designs as opposed to physical improvements in the work environment, such as reconfiguring the structural layout of the ICU room, which might be cost prohibitive.

### Conclusions

This WDA provided several important insights for understanding nurse decision-making about patient mobility within the context of the ICU work environment and identified opportunities for technology design to support decision-making. The results of this study identify strategies for integrating critical patient and unit-level information to inform patient mobility decisions. Future studies should investigate the acceptability and feasibility of interventions to support nurse decision-making about mobility interventions. This analysis demonstrates that interdependencies exist between patients, nurses, other members of the health care team, and unit resources. Therefore, multicomponent interventions that address constraints in the work environment, such as lack of human resources and nurse workload, are needed. Systems-based approaches to improve delivery of mobility and patient outcomes must include interventions that are based upon how nurses make these complex decisions within the work environment.

CWA is a valuable framework for understanding the environment in which ICU nurses make patient mobility decisions and for identifying priorities to target with future CDS designs for supporting mobility decisions at the point of care. Findings from this study provide an understanding of how mobility decisions are made from the perspective of the ICU work environment and identify a need to consider the types of information nurses use to make knowledgeable and efficient patient mobility decisions.

## Appendix

Multimedia Appendix 1 Interview guide.

## Abbreviations

CDS: clinical decision support
CWA: cognitive work analysis
EHR: electronic health record
ICU: intensive care unit
PT: physical therapist
RN: registered nurse
WDA: work domain analysis
