# Supplementary material for: Supporting Decision-Making About Patient Mobility in the Intensive Care Unit Nurse Work Environment: Work Domain Analysis
Source: JMIR Nurs. 2022 Sep 27;5(1):e41051. doi: 10.2196/41051 (PMC9555320; doi:10.2196/41051)
Supplement: Multimedia Appendix 1 [file nursing_v5i1e41051_app1.docx]

**ID#:** __________

Interview Guide

Thank you for participating in this study today. I would like to audio-record the interview to help me capture your responses. May I record the interview?

During the interview, please use only first names and no patient names. This will help me keep your responses private. Your answers will not be individually reported to your colleagues or supervisor.

The interview will take about 60 minutes to complete. If you need to take a break during the interview, please let me know and I will pause the interview.

If any of my questions aren’t clear please let me know and I will rephrase the question for you. Please remember that you are not required to answer any specific question. You may also leave the interview at any time.

Do you have any questions before we start the interview?

Initial interview questions may consist of the following:

1. Often nurses have different ideas of what is considered mobility for patients. Can you describe for me how you define patient mobility in the ICU? (timing, frequency, etc)
2. When you think about patient mobility, what do you think is the nurses’ role? (coordination, timing, barriers)
3. I'd like you to think about the last few weeks when you have worked. Can you describe for me any activity you engaged patients in that you would identify as early mobility?

Did you need help from others? Who?

Why did you do this?

How did you engage the patient in the activity? What was the outcome?

Can you describe for me all the things you had to do before you got the patient up?

What would have happened if that patient hadn’t mobilized during your shift?

1. I’d like you to think of a particularly complex patient case or situation. Can you describe for me any activity in that situation that you would identify as early mobility?

Can you describe for me all the things you had to do before you got the patient up?

What information did you use to lead you to that outcome?

What would have happened if that patient hadn’t mobilized during your shift?

1. I’d like you to think of a patient that you didn’t mobilize, but you thought they would benefit. What got in the way? What led you to that outcome?
2. Describe for me a type of patient that you would not get out of bed, how about a patient that you would not walk (patient characteristics - physical, cognitive, and psychosocial)?

How would these patients have activity advanced? By whom?

What information did you use to lead you to that outcome?

What would have happened if that patient had mobilized during your shift?

1. Is there anything else you would like to add that I didn’t ask about?

Follow up questions may include:

1. What/why/how
2. Can you describe for me your thoughts on mobilizing the patient in the situation you just described?
3. I want you to think about the last situation you described. Can you describe for me all the things you had to do before you got the patient up?
4. I want you to think about the last situation you described. What would have happened if that patient hadn’t mobilized during your shift?
